# In This Issue: Volume 116, Issue 9, September 2025

**DOI:** 10.1111/cas.70177

**Published:** 2025-09-01

## How Do Cancer Cells Create Cancer‐Associated Fibroblast Subtypes? Impacts of Extracellular Vesicles on Stromal Diversity

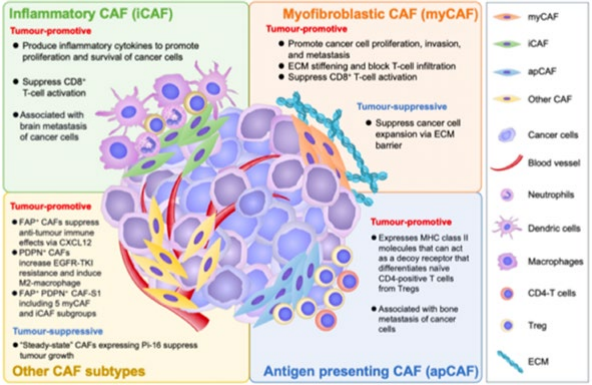

Cancer doesn't just grow; it changes the environment around it to help it spread and resist treatment. To thrive, tumors reshape their surroundings, turning nearby healthy cells into accomplices. This surrounding network, known as the tumor microenvironment (TME), is a complex ecosystem made up of immune cells, blood vessels, and structural support cells. One important player in the TMEs is a group of support cells called cancer‐associated fibroblasts (CAFs). These cells help build the structure around tumors, regulate immune responses, promote blood vessel growth, and support cancer cell survival. However, not all CAFs are the same—Some help tumors grow, while others might slow them down.

In this review, Yutaka Naito explores the question: how do cancer cells create different types of CAFs? Recent research shows that cancer cells use tiny packages called extracellular vesicles (EVs)—couriers of molecular cargo—to send instructions to nearby fibroblasts. These vesicles carry proteins, RNAs, and other signals that can transform normal fibroblasts into different CAF subtypes.

The variety, or heterogeneity, of CAFs makes it harder to treat cancer. Therapies that target one subtype might miss others, or even worsen the situation. That's why understanding how cancer cells ‘educate’ fibroblasts through EVs is so important. If scientists can figure out how to block or reverse these signals, they might be able to stop tumors from recruiting harmful CAFs, or even program them to help the body fight back.

This review paper highlights the need for future cancer treatments to consider not just the cancer cells, but also the complex community of cells in the TME—especially the CAFs that cancer can turn into its allies.

https://onlinelibrary.wiley.com/doi/full/10.1111/cas.70133

## Selinexor Reduces the Immunosuppression of Macrophages and Synergizes With CD19 CAR‐T Cells Against B‐Cell Lymphoma

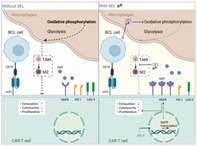

Chimeric antigen receptor (CAR)‐T cell therapy is a cutting‐edge cancer treatment that involves collecting a patient's own immune cells, genetically modifying them to recognize cancer, and then reinfusing them to attack the tumor. It has been a game‐changer for some blood cancers like B‐cell lymphoma, but many patients don't respond fully or experience results that are not long‐lasting.

This is because the cancer finds ways to fight back. It can evade immune detection and create a hostile tumor microenvironment filled with suppressive immune cells—such as **M2‐type macrophages**—that weaken or disable the CAR‐T cells meant to destroy it.

In this study, researchers explored whether **selinexor**, an existing cancer drug, could help improve the effectiveness of CAR‐T cell therapy. Selinexor blocks a protein called **XPO1**, which exports various proteins from the cell nucleus to the cytoplasm. Using laboratory and animal models that mimicked the tumor's complex environment, the researchers found that selinexor **reduced the number of immunosuppressive M2 macrophages**, helping to create a tumor microenvironment that was more supportive of immune activity. Notably, when selinexor was administered **before CAR‐T cell infusion**, it helped reduce CAR‐T cell exhaustion, boosted their ability to multiply, and enhanced their capacity to kill tumor cells.

While higher doses of selinexor led to side effects such as inflammation and weight loss in animal models, lower doses were both safe and effective. Importantly, selinexor did not alter the expression of CD19—the key marker recognized by CAR‐T cells—indicating that it did not interfere with their ability to target cancer cells. At the same time, selinexor made the tumor microenvironment more supportive of immune activity by reducing suppressive M2 macrophages.

Overall, this research demonstrates that selinexor, a small‐molecule drug, can reduce immunosuppressive M2 macrophages and enhance the function and proliferation of CAR‐T cells in lymphoma models. These findings provide a strong rationale for combining selinexor with CAR‐T therapy to develop more effective and safer treatment strategies, which may benefit lymphoma patients.

https://onlinelibrary.wiley.com/doi/full/10.1111/cas.70123

## PRRX2 Regulates GLI2 to Promote Proliferation, Invasion, and Metastasis by Inhibiting Senescence via Hedgehog Signaling

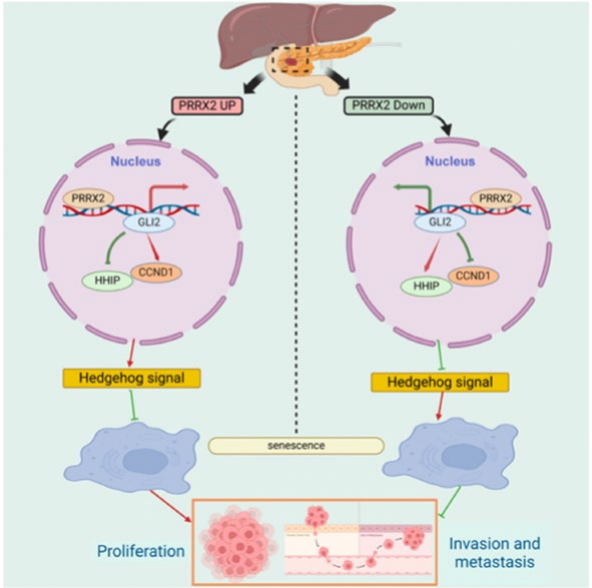

Pancreatic cancer (PC) is one of the most aggressive and difficult‐to‐treat cancers, with a high rate of metastasis, resistance to therapy, and poor early detection. As a result, it remains a leading cause of cancer‐related death. Moreover, only a small percentage of patients are eligible for surgery at diagnosis, underscoring the need for novel biomarkers to track the disease's development and progression.

While ‘senescence’ or cellular aging—a state of stable growth arrest—has been reported to suppress PC progression, the underlying molecular mechanisms remain elusive.

In this issue, Huang et al. examined the role of paired‐related homeobox gene 2 (*PRRX2*), which encodes a transcription factor associated with senescence in PC progression. While *PRRX2* has been widely implicated in the progression and metastasis of colon, gastric, and prostate cancers, its role in PC development remains unexplored.

The researchers analyzed the gene expression profiles of PC tissue samples using The Cancer Genome Atlas. They observed a significantly higher level of *PRRX2* expression in PC tissues, which correlated with decreased disease‐free survival. Moreover, PC tissues showed an elevated level of *PRRX2* compared to nearby non‐tumor tissues.

Delving deeper, the team found that suppressing the expression of *PRRX2* inhibited the growth of PC cells and markedly reduced their invasive and metastatic potential. Furthermore, mice injected with cells deficient in *PRRX2* formed smaller tumors and exhibited fewer and smaller metastases. Notably, cells deficient in *PRRX2* showed increased levels of markers associated with senescence and cell cycle arrest.

To further elucidate the mechanistic role of *PRRX2* in PC, the researchers analyzed genes and signaling pathways that were co‐expressed/activated with *PRRX2* in PC. They found that PC tissues showed high expression of *GLI2*, a key target in the Hedgehog signaling pathway. Notably, suppression of *PRRX2* led to a significant decrease in *GLI2* expression.

Further, the researchers demonstrated that *PRRX2* directly binds to the promoter region of *GLI2*, promoting its expression and activating Hedgehog signaling (a key developmental signaling pathway). Conversely, inhibition of GLI2 suppressed downstream targets and reduced the proliferation, invasion and metastatic potential of *PRRX2*‐overexpressing cells. Similar effects were observed when these cells were treated with a senescence‐inducing drug.

Together, these findings suggest that *PRRX2* suppresses senescence in PC cells by upregulating *GLI2* and activating Hedgehog signaling, thereby, making them aggressively malignant. Given the pivotal role of senescence in stalling PC progression, targeting *PRRX2* may offer a promising therapeutic strategy.

https://onlinelibrary.wiley.com/doi/full/10.1111/cas.70134

